# Expect the Unexpected: A Rare Case of Isolated Superior Mesenteric Artery Vasculitis

**DOI:** 10.7759/cureus.40106

**Published:** 2023-06-07

**Authors:** Aseel Alkhader, Nourhan Saleh, Mahmoud M Mansour, Omar Hussein, Baraa Saad

**Affiliations:** 1 Internal Medicine, Faculty of Medicine, Jordan University of Science and Technology, Irbid, JOR; 2 Internal Medicine, Faculty of Medicine, Alexandria University, Alexandria, EGY; 3 Geriatrics, University of Missouri School of Medicine, Columbia, USA; 4 Sleep Medicine, University of Missouri Kansas City School of Medicine, Kansas City, USA; 5 Infectious Diseases, University of Texas Health Science Center, Galveston, USA

**Keywords:** inflammation, abdominal pain, vasculitis, intestinal ischemia, superior mesenteric artery

## Abstract

Vasculitis of the mesenteric vessels is considered rare and typically occurs as a part of systemic inflammation. Isolated mesenteric artery vasculitis without systemic involvement is rarely reported in the literature. Clinical presentation is usually nonspecific which can range from abdominal pain, nausea and vomiting to gangrene and intestinal perforation in severe cases. Recognizing mesenteric artery vasculitis as a potential cause of abdominal pain can be challenging, and delay in diagnosis can lead to significant mortality and morbidity. Herein, we present a case of a 19-year-old male who initially presented with abdominal pain. Later, isolated superior mesenteric artery (SMA) vasculitis was confirmed by CT angiography. Treatment with systemic steroids alone resulted in a marked improvement in the patient’s symptoms as well as in radiographic findings.

## Introduction

Vasculitides are a group of diseases that are characterized by blood vessel wall inflammation. Vasculitis is considered rare; it affects 20 individuals per million each year, and approximately half of the vasculitis cases involve mesenteric arteries, with 16% of those cases being isolated mesenteric vasculitis [1]. Among these conditions that may involve the mesenteric vessels as part of systemic inflammation are polyarteritis nodosa (PAN), granulomatosis with polyangiitis (GPA), eosinophilic granulomatosis with polyangiitis (EGPA), microscopic polyangiitis (MPA), IgA vasculitis and Takayasu arteritis [2].

Isolated mesenteric vasculitis poses a diagnostic challenge, and delay in therapy is associated with significant morbidity and mortality due to intestinal ischemia resulting in necrosis, bleeding or perforation [3]. In this report, we present a rare case of isolated superior mesenteric artery (SMA) vasculitis that was successfully diagnosed and managed medically with corticosteroids. This article was previously presented as a meeting abstract at The 2022 Midwest Clinical and Translational Research Meeting on March 04, 2022.

## Case presentation

A 19-year-old Caucasian male with no known past medical history presented to the emergency department with epigastric pain for three months that worsened a week before his presentation. The pain was burning, non-radiating, intermittent and exacerbated after eating. He denied other gastrointestinal symptoms of melena, hematochezia or hematemesis. Additionally, he denied fevers, night sweats or weight loss as well as joint pain, swelling, skin rash or mouth ulcers. He denied alcohol, smoking or illicit drug use. There was no known recent viral illness or vaccination prior to the presentation. Family history was negative for gastrointestinal tumors or rheumatological disorders.

On physical exam, his vital signs were stable with a blood pressure of 121/73 mmHg, heart rate of 71 beats per minute, respiratory rate of 16 breaths per minute and body temperature of 36.8°C. Oxygen saturation was 99% on room air. He had mild epigastric tenderness, but no arterial murmurs were noted on abdominal auscultation. Initial laboratory workup including complete blood count, comprehensive metabolic panel and serum lipase were within normal limits.

**Figure 1:**
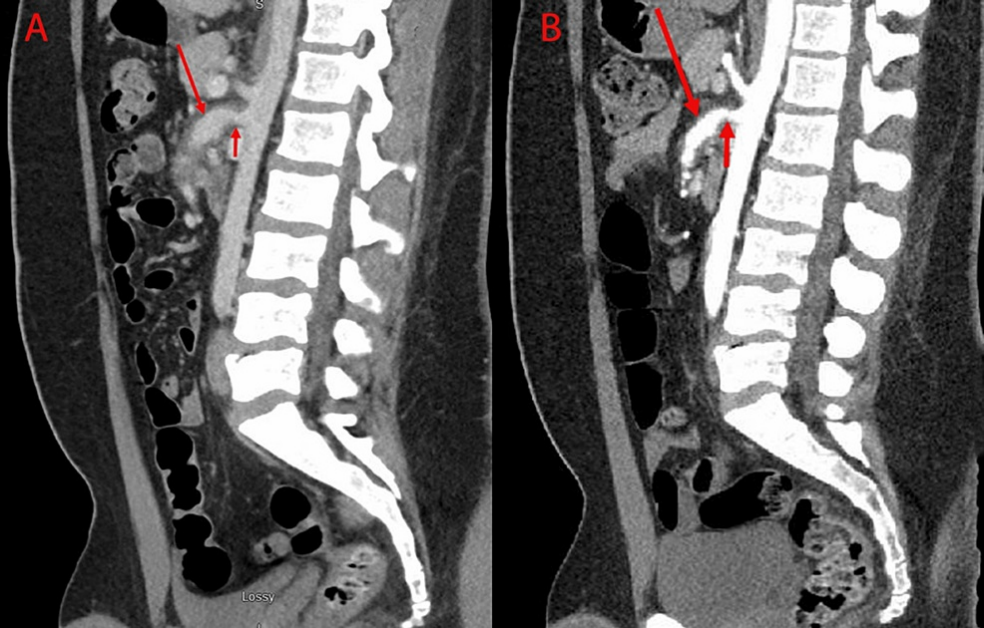
CT scans showing the sagittal sections of the superior mesenteric artery. CT scans showing the sagittal sections of the superior mesenteric artery before (A) and after (B) two months of treatment with steroids. A: CT scan with intravenous contrast showing the narrowing of the superior mesenteric artery (short arrow) followed by 1.5 cm aneurysmal dilation (long arrow). Notice the increased stranding (inflammation) of the vessel wall. B: CT angiography scan two months after treatment with steroids showing slightly improved narrowing of the superior mesenteric artery (short arrow) and aneurysmal dilation (measuring now 1.3 cm, long arrow). Notice the improved stranding (inflammation) of the vessel wall.

A computed tomography (CT) scan of the abdomen with intravenous contrast revealed a 4 mm stricture at the origin of the superior mesenteric artery (SMA) followed immediately by a 1.5 cm aneurysmal dilation. This dilation spanned 5 cm and was associated with fat stranding around the artery wall (Figure 1A). These findings were concerning for medium vessel vasculitis.

The patient was admitted to the hospital where extensive evaluation for etiologies of vasculitis and signs of systemic involvement was performed. This workup showed elevated erythrocyte sedimentation rate (ESR) at 25 mm/hr (normal: ≤15 mm/hr for males <50 years) and C-reactive protein (CRP) of 1.5 mg/dL (normal: < 1.0 mg/dL); antinuclear antibodies (ANA), antineutrophil cytoplasmic antibodies (ANCA), myeloperoxidase (MPO) antibodies, anti-Pr-3 antibodies, complement levels and cryoglobulins were all within normal ranges; and viral hepatitis panel, human immunodeficiency virus (HIV) and urine drug screen were also negative.

The urinalysis was negative for microscopic hematuria and proteinuria. Antiphospholipid, cardiolipin and beta-2-glycoprotein antibody tests were negative. Additionally, CT angiography of the chest showed no evidence of vasculitis in medium or large vessels of the thorax. With all the aforementioned tests being negative, a diagnosis of isolated SMA vasculitis was presented.

Surgical intervention was deferred due to the relatively small size of the SMA aneurysm and lack of signs of bowel edema or ischemia on imaging. The patient was started on high-dose corticosteroids with prednisone 60 mg daily. Over a two-week period, he reported improvement in his abdominal pain. Two months after his initial presentation, a repeat CT angiogram showed a resolution of the inflammation surrounding the SMA and a reduction of the aneurysm size to 1.3 cm in cross-sectional diameter (Figure 1B). Following these stable CT findings, the prednisone dose was progressively tapered over a period of four months until its cessation. The patient was subsequently followed with a surveillance CT scan that showed a stable size of the SMA aneurysm two years after his presentation.

## Discussion

Isolated mesenteric vasculitis, involving vessels of the gastrointestinal tract without the involvement of other organ systems, is rare, and data regarding this entity are limited [4]. Chronic inflammation can lead to weakening of the vessel wall causing an aneurysm or wall thickening, resulting in stenoses. The underlying etiology of isolated mesenteric vasculitis is still unknown. One theory suggests that local exposure to antigens or infectious agents may lead to a local immune response. Other recent reports have found that viral infections, like COVID-19, may trigger inflammatory processes that could contribute to mesenteric vasculitis [5].

Clinical manifestations of mesenteric vasculitis are usually attributed to mesenteric ischemia. The most common presenting symptom is abdominal pain; patients might also have nausea, vomiting and rectal bleeding. In severe cases, intestinal perforation or gangrene can lead to altered mental status and hemodynamic instability [6]. A diagnosis based solely on nonspecific gastrointestinal symptoms is difficult. However, the condition should be considered in young patients who are presenting with symptoms of mesenteric ischemia without risk factors of atherosclerotic disease [2].

A diagnosis of isolated mesenteric vasculitis requires imaging studies or histopathological examination of surgical specimens. Conventional catheter-based angiography has been used to diagnose most reported cases. More recently, CT or MR angiography is being increasingly utilized to evaluate medium and large vessel vasculitis and may show circumferential arterial wall thickening, luminal narrowing and aneurysms [7].

Effective treatment of isolated mesenteric vasculitis requires close collaboration between surgical and medical specialties. Surgery and/or endovascular treatment might be required for perforations, fistulas, ischemia or severe stenoses and aneurysms unresponsive to medical therapy. Higher rates of complications have been reported with endovascular treatment including restenosis, thrombosis and bleeding; hence, it is usually reserved for the aforementioned major vascular complications [1,8]. Systemic glucocorticoids are the cornerstone of medical treatment for isolated mesenteric vasculitis, whereas systemic vasculitis usually requires the addition of other immunosuppressive therapies [9].

## Conclusions

Isolated mesenteric vasculitis is rare and poses a diagnostic challenge due to nonspecific presentation. We described a rare case of isolated superior mesenteric artery vasculitis which was diagnosed with a CT angiogram. Treatment with systemic steroids resulted in significant improvement in patient's outcomes. This case highlights that a high index of suspicion for SMA vasculitis is key for timely diagnosis and management which is needed to avoid potentially life-threatening complications.

## References

[1] Rits Y, Oderich GS, Bower TC (2010). Interventions for mesenteric vasculitis. J Vasc Surg.

[2] Salvarani C, Calamia KT, Crowson CS (2010). Localized vasculitis of the gastrointestinal tract: a case series. Rheumatology (Oxford).

[3] Soowamber M, Weizman AV, Pagnoux C (2017). Gastrointestinal aspects of vasculitides. Nat Rev Gastroenterol Hepatol.

[4] Atisha-Fregoso Y, Hinojosa-Azaola A, Alcocer-Varela J (2013). Localized, single-organ vasculitis: clinical presentation and management. Clin Rheumatol.

[5] Narwan A, Sauer A, Talwar T, Willes O, Ranasinghe N, Ranasinghe L (2022). A rare presentation of SMA vasculitis with chest and upper back pain: case report. Asp Biomed Clin Case Rep.

[6] Gnanapandithan K, Sharma A (2022). Mesenteric vasculitis. StatPearls [Internet].

[7] Kakehi E, Adachi S, Fukuyasu Y (2019). Superior mesenteric artery vasculitis in Behçet's disease: a case report and literature review. Intern Med.

[8] Saadoun D, Vautier M, Cacoub P (2021). Medium- and large-vessel vasculitis. Circulation.

[9] Mohan N, Gomes MN, Cupps TR (2002). Isolated superior mesenteric artery vasculitis with response to glucocorticoids. J Clin Rheumatol.

